# Do gender and torus mandibularis affect mandibular cortical index? A cross-sectional study

**DOI:** 10.1186/1746-160X-3-37

**Published:** 2007-10-30

**Authors:** Serdar Uysal, Berna L Çağırankaya, Müjgan Güngör Hatipoğlu

**Affiliations:** 1 Hacettepe University Faculty of Dentistry, Department of Oral Diagnosis and Radiology, Sihhiye, Ankara, Turkey; 2 Hacettepe University Faculty of Dentistry, Department of Oral Diagnosis and Radiology, Sihhiye, Ankara, Turkey; 3 Dumlupinar University Research and Training Hospital, Dental Clinic, Kutahya, Turkey

## Abstract

**Background:**

The interactions between torus and several factors such as age, gender, and dental status have not been studied comprehensively. The purpose of this study was to determine the effect of gender on the mandibular cortical index (MCI) and to investigate a possible association between torus mandibularis (TM) and MCI.

**Methods:**

The study consisted of 189 consecutive patients referred to Department of Oral Diagnosis and Radiology of Hacettepe University within 30 workdays. Patients who did not have systemic disorders affecting bone density were included; and the age, gender, dental status and existing TM of the patients were recorded. Morphology of the mandibular inferior cortex was determined according to Klemitti's classification on panoramic radiographs.

**Results:**

MCI was affected by age and gender (*P *< 0.05). No significant relationship was found between TM and MCI (*P *> 0.05).

**Conclusion:**

In the study population, MCI was affected by age and gender. As age increased, semilunar defects could be seen on the cortex of the mandible and MCI values increased. Women appeared to have higher MCI values than men.

## Background

Increase in the porosity of bone coupled with decrease in bone density and minor loss of bone starts in the 30's of humans [1]. Good skeletal mineral status is related to physical and muscular activity [2], and bone mineral density (BMD) may be considered as an essential component of bone quality [3]. In the context of the masticatory system, mineral loss in the mandibular cortex depends on the rate of mineral loss in the skeleton and age [3]. BMD can be evaluated by techniques such as computed tomography [4] and dual energy x-ray absorbsiometry (DEXA) [5]. However, these techniques are expensive and therefore, have not been considered applicable in all situations [5,6].

Because the radiographic appearance of the jaws change in osteoporotic patients, the relationship between the mandibular morphology and the rate of osteoporosis can be quantified by the determination of the thickness and completeness of mandibular inferior cortex. This may provide the opportunity of early identification of osteoporotic patients, who actually need treatment [5]. In this regard, the use of indexes could be considered as a simpler and cost-effective approach for detecting osteoporosis. With the use widely-used indexes in dentistry, such as Mandibular Cortical Index (MCI) and Mandibular Index (MI) on panoramic radiographs, osteoporosis may be detected at early stages [5,7]. The simplest method to determine the bone quality is MCI, which is a simple method based on the classification of radiographic appearance of the mandibular inferior cortex [8-10].

Research has shown that bone metabolism in the alveolar process alters markedly upon tooth extraction, and the loss of tooth influences the prevalence of torus [11,12]. In addition, the number of functioning teeth appears to be an important factor on the presence of torus [13]. So far, the interactions between torus and several factors such as age, gender, and dental status have not been studied comprehensively [14]. The aim of this study was, therefore, to determine the effect of gender on MCI and whether any relationship exists between torus mandibularis (TM) and MCI.

## Methods

The study consisted of 189 consecutive patients referred to Department of Oral Diagnosis and Radiology of Hacettepe University within 30 workdays. Informed consent was obtained from patients after explaining the study protocol. Both the consent form and the study protocol were performed upon approval by the Institutional Human Subject Review Committee of Hacettepe University (approval number: HEK 07/123-8).

The inclusion criterion stipulated selection of patients who only needed panoramic radiographic examination for the purpose of routine dental diagnosis and treatment planning. Patients who had systemic disorders that could affect bone density were excluded. Thus, none of the selected 189 patients were known to have endocrine, metabolic or skeletal disorders or any local pathology that could affect MCI or TM. All the panoramic radiographs were diagnostically acceptable for the evaluation of MCI.

Panoramic radiographs of the patients were obtained by an Orthopantomograph (OP100^®^, Instrumentarium Corp., Finland) which had a magnification value of 1.3. No further magnification correction was undertaken during evaluation of the radiographs. The head of the patients were positioned so that the line from the tragus to the outer canthus was parallel to the floor, and the antero-posterior position of the patients was achieved by having patients place the incisal edges of their maxillary and mandibular incisors into the bite block. All films were processed in an automatic processor (XR 24, Dürr Dental GmbH & Co.KG, Bissingen, Germany). Panoramic radiographs with diagnostic contrast and density, and absence of positioning errors were evaluated by one of the two observers (B.Ç. or S.U.). 12 radiographs, which did not conform to these criteria, were excluded. A pilot study was performed to evaluate intra and inter-observer agreement with Kappa statistics, and was determined to be good (66%) and excellent (86%), respectively.

The morphology of mandibular inferior cortex was determined by observing both sides of the mandible distally from the mental foramen using Klemitti's classification [8];

C1: The endosteal margin of the cortex is even and sharp on both sides,

C2: The endosteal margin shows semilunar defects (resorption cavities) with cortical residues 1 to 3 layers thick on one or both sides,

C3: The cortical layer contains heavy endosteal cortical residues and is clearly porous.

A standard form was prepared to record the age and gender of the patient, and torus mandibularis, if detected. The existence of TM was recorded upon verification by visual inspection and digital palpation. Bone processes, which could be felt by palpation but not by inspection, were not considered as TM.

The dentition was classified as full, partial or edentulous (excluding the third molars). Patients were accepted as partially dentate in the absence of premolar or molar teeth in the left or right sides of the mandible. They were also accepted as partially dentate when occlusion with the opposing arch could not be achieved due to the lack of maxillary teeth, even in the presence premolar and molar teeth on the mandible.

All data were analyzed using Statistical Package for the Social Sciences (SPSS) V.11.5 (SPSS Inc. Chicago, IL, USA). Cross-tabulations and Chi-square statistics were computed with the statistical significance set at *P *< 0.05 [15].

## Results

119 female (63%) and 70 male (37%) patients with a mean age of 45.71 years (range: 21–86) were included. There was no racial or ethnic diversity within the study population and all participants were Caucasians. 101 patients (53.4%) had no systemic disorders, while 88 patients (46%) had systemic disorders which did not affect bone mineral density.

Seven patients (3.7%) had TM on the right side, 3 patients (1.6%) on the left side, and 13 patients (6.9%) had bilateral TM. 166 patients did not have TM. No significant relation was found between TM and MCI (*P *> 0.05). Among patients having TM, 8 patients (13.1%) were C1, 14 patients (12.5%) were C2 and only one patient (6.3%) was C3. No significant relationship could be found between TM and dental status of the patients (*P *> 0.05). TM was detected in 10% of men and 13.4% of women. TM was diagnosed in 16.7% (n = 12) of the 21–40 age group, 8.7% (n = 8) of the 41–60 age group, and 12% (n = 3) of the 60+ group.

13% of the total population had full dentition but 14% of them had edentulousness. For the mandible, 22% of the patients had full dentition but 15% of them had edentulousness. MCI was significantly affected by the status of dentition (x^2 ^= 16.419, p = 0.0001). Most patients who were C1 (82% within MCI) had full occlusion and patients who were C3 (62% within MCI) had edentulousness.

**Figure 1:**
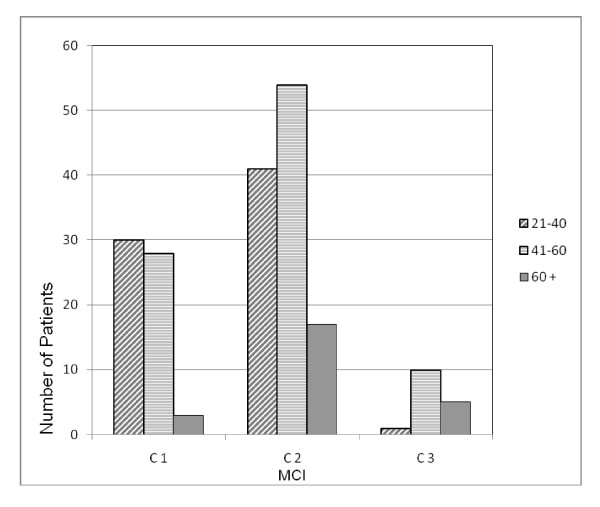
Patients MCI distribution according to age groups.

MCI was distributed as follows: C1 = 61 patients (32.3%), C2 = 112 patients (59.3%) and C3 = 16 patients (8.5%). Three subgroups based on age were: A. 21–40 (72 patients, 38.1%), B. 41–60 (92 patients, 48.7%), and C. 60+ (25 patients, 13.2%). The rationale of using subgroups was to provide comparative analysis of relatively young and old patients. Cross-tabulation of MCI by age demonstrated an age-related pattern. While age increased, C1 decreased (x^2 ^= 14.457, p = 0.006), (Figure 1).

**Figure 2:**
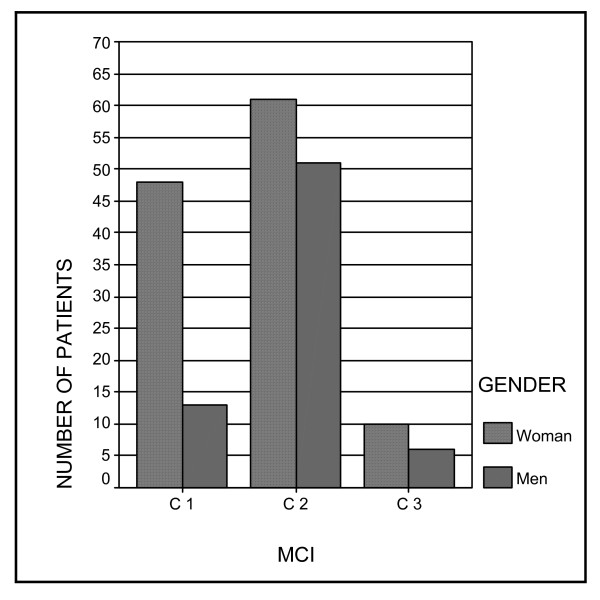
Patients MCI distribution according to gender.

Cross-tabulation of MCI by gender demonstrated a gender-related pattern. C1 and C3 was significantly higher in women than in men (x^2 ^= 9.939, p = 0.007), (Figure 2). 78.7% of the C1 group was women (n = 48) and 21.3% was men (n = 13). 62.5% of the C3 was women (n = 10) and 37.5% was men (n = 6).

## Discussion

Although an extensive search of the literature shows that a possible association between TM and MCI has not been evaluated elsewhere, the association between age, gender and MCI is not a new finding. On the other hand, the present study provides information with regard to the association between age, gender and MCI in Turkish population for the first time. Since this study was designed as a single-centre study, it does not represent the entire Turkish population. Moreover, the small sample size, strict inclusion criteria (those who only needed panoramic radiographs), short duration of patient selection, and the few number of patients with TM might be considered as shortcomings of the present study. However, even within these limitations, the study has provided significant findings.

Bone mineral density (BMD) is an important component of bone quality. It has been measured by several techniques including quantitative computed tomography (QCT), single or dual x-ray absorbsiometry (SXA or DEXA) and quantitative ultrasound (QUS) [4,5]. However, these techniques are very expensive [5,6], for which the development of more cost-effective and equally-reliable alternatives may be beneficial.

It has been stated that low BMD values are related with high MCI values (C3), which can be extrapolated to clinical practice [4-6,8,10,16,17]. It has been shown that BMD values measured by DEXA were related to MCI [18]. An increase in the number of people with C3 was observed as they aged. This is probably due to bone loss that develops with increasing age [16].

Panoramic radiography is a routine imaging method in dentistry. As changes in the mandibular cortex can be detected on the panoramic radiograph of patients with osteoporosis, panoramic radiograph can be considered an invaluable diagnostic tool for dentists [7]. Provided that diagnostic values are not lost due to projection errors resulting from disposition of the head [8], panoramic radiographs can be used in determining the bone density, as a relationship between mandibular bone mineral density and the skeletal areas in evaluating osteoporosis has been shown [4,14]. MCI is a simple, non-numerical method to classify the radiographic image of the mandible [8-10,18]. It has been reported that panoramic radiographs could be useful for identifying women with low BMD or osteoporosis [4,10]. On the other hand, a number of studies suggest that osteoporosis cannot be diagnosed on panoramic radiographs [8], and recommend dentists to refer postmenopausal women with eroded cortex for bone densitometry [19].

Provided that MCI is to be used in identifying the prevalence of osteoporosis in epidemiologic studies, the calibration of the observers is essential [18,20]. The appropriateness of utilizing MCI [9] and its repeatability has been documented [16]. It has also been shown that inter-observer agreement often appears to be exact or perfect [6]. In the pilot study, intra and inter-observer agreement with kappa statistics was determined as good (66%) and excellent (86%), respectively.

Identifying the quality of bone is essential in planning advanced treatment options such as dental implants, and in diagnosing patients with osteoporosis. Halling et al. [21] demonstrated that assessment of mandibular cortex patterns is a reliable method to exclude osteoporosis. Patients having positive findings related to MCI should be evaluated further for potential risk of osteoporosis. Therefore, dentists may be able to use this negative predictive value as a possibility for excluding large populations from unnecessary DEXA screening.

In the present study, MCI was significantly affected by the status of dentition. Most patients who were C1 (82% within MCI) had full occlusion and patients who were C3 (62% within MCI) had edentulousness. Partially dentate patients appeared to have higher MCI values. The lack of full occlusion causes insufficient occlusal forces projected to the mandible, which may affect the mandibular cortex, resulting in higher MCI values (C3).

Knezovi-Zlatari et al. [22], showed that C3 was more frequently observed in patients due to age distribution, and that there was a significant increase in the incidence of elderly female patients with C3. In our study, C3 was significantly higher in women than in men. 61.9% of partially dentate patients was women (n = 26), which maybe the reason why C3 was significantly higher in women.

In the present study, all three types of MCI was observed. C3 was observed in the eldest age group (60+). With increasing age, the incidence tooth loss increased and forces that would influence the mandibular bone decreased. This may probably account for the higher MCI values in eldest age group. Our findings are not in agreement the literature which showed that TM is found more commonly in men than in women [23-25]. TM was detected in 10% of the men and 13.4% of women. TM was most frequently seen in the 21–40 age group and 73.6% of this age group consisted of women, which could explain the gender difference. TM are frequently observed in young adults and in middle-aged persons [24,25]. Similar to this finding, 16,7% of the 21–40 age group and 8.7% of the 41–60 age group were found to have TM. Our study group did not include patients less than 21 years of age, for which a comparison with younger patients could not be made with older ones. Our results confirm that TM can be seen in throughout lifetime [26]. Our study did not aim to search for the time which TM was first observed in patients. Thus, we could not report on data which could be indicative of the reason for TM formation. It has been stated that as TM could be seen in the middle phase of the life, which indirectly suggests not only a genetic cause, but also environmental and functional factors related to the effect of masticatory stress on the formation of TM [25]. Thus, the number of existing neighboring teeth seemed to be a significant factor for the survival of tori [13].

Among other variables investigated, the forces applied on the mandible appeared to be influenced by the number of teeth. The study by Eggen and Natvig supports the postulation that functional forces significantly ifluence the incidence of torus [13], and that the frequency of TM decreases with increasing tooth loss. Thus, the number of functioning teeth is an important factor for existence [13], and the prevalence [12] of TM. Ossenberg suggested that although both genetic and environmental factors may play a role in the formation of torus, the masticatory system should be considered as the primary essential initiative factor [13,27]. Kerdporn and Sirirungrojying [28] also found a strong association between the presence of TM and occlusal stress. In a study by Clifford et al. [29], TM has been reported as a result of parafunctional activity. They suggested that TM might be a useful marker of past or present parafunctional activity for some patients. The prevalence of TM and parafunctional activity has been found to be higher in patients with temporomandibular disorder [29]. Hence, TM might be useful as an indicator of increased risk of temporomandibular disorder [30]. Cagirankaya et. al. [31] showed that subjects with TM seems to have higher bite force than those without TM. The association between formation of tori and parafunction in the form of bruxism has been supported by the results of Eggen and Natvig [13]. Eggen [32] evaluated the etiology of TM in a group of bruxist patients. The bruxist group showed heavy muscular forces leading to occlusal stress. The author concluded that the etiology of TM was 30% of genetic origin, while 70% of patients were affected environmentally, i.e., by occlusal stress.

A significant positive correlation between the presence of TM and BMD has been reported, and the presence of TM appears to be an indicator of denser skeletal mass and bone density [33]. The presence of tori at young adulthood may be a marker of higher BMD in the future, and of a lower risk for developing osteoporosis [33]. Hosoi et al. [34] found a significant positive correlation between the presence of palatal tori and BMD at the femur and radius. On the other hand, they could not find a significant correlation between mandibular tori and BMD at the radius. They mentioned that their results are suggestive of some common mechanisms that are involved in the elevation of skeletal BMD and the occurrence of oral exostoses. Padbury et al. [35] found a high incidence of tori in patients with primary hyperparathyroidism, and explained their findings with the biomechanical forces particular to the oral cavity, cortical bone loss and trabecular expansion.

TM has been shown to indicate higher bone density [33] and MCI has been related to BMD [4-6,8,10,16,17]. Existence of TM can be a useful sign of higher bone density and lower MCI values. In the present study, however, no significant associations were found between TM and dental status, and between TM and MCI. The limited sample size in our study might be a reason why our results did not support the hypothesis that TM is affected by dental status. Moreover, the study plan did not involve investigation of a possible relationship between TM and dental status.

Within the limitations of the present study, the following conclusions were drawn:

1. MCI can be used in evaluating the quality of bone, as it is easy to apply and is relatively cost-effective. Moreover, unnecessary DEXA screening can be avoided, as patients will be advised to visit a doctor when they are diagnosed of having osteoporosis risk by panoramic radiographs.

2. MCI was affected by gender and age. With increasing age, women showed more porosity on mandibular cortex, as the mandibular cortex becomes more porous. When the effects of age and gender are evaluated together, women may be expected to have more porous mandibular cortex (higher MCI values).

3. Our results failed to establish an association between TM and MCI, which could be due to the limited sample size. Further studies on larger populations will be necessary to investigate a possible association between TM and MCI.
